# *Clostridium difficile* ribotype 017 – characterization, evolution and epidemiology of the dominant strain in Asia

**DOI:** 10.1080/22221751.2019.1621670

**Published:** 2019-05-29

**Authors:** Korakrit Imwattana, Daniel R. Knight, Brian Kullin, Deirdre A. Collins, Papanin Putsathit, Pattarachai Kiratisin, Thomas V. Riley

**Affiliations:** a School of Biomedical Sciences, The University of Western Australia, Crawley, Australia; b Department of Microbiology, Faculty of Medicine Siriraj Hospital, Mahidol University, Bangkok, Thailand; c School of Veterinary and Life Sciences, Murdoch University, Murdoch, Australia; d Department of Molecular and Cell Biology, University of Cape Town, Cape Town, South Africa; e School of Medical and Health Sciences, Edith Cowan University, Joondalup, Australia; f PathWest Laboratory Medicine, Queen Elizabeth II Medical Centre, Nedlands, Australia

**Keywords:** *Clostridium difficile*, ribotype 017, epidemiology

## Abstract

*Clostridium difficile* ribotype (RT) 017 is an important toxigenic *C. difficile* RT which, due to a deletion in the repetitive region of the *tcdA* gene, only produces functional toxin B. Strains belonging to this RT were initially dismissed as nonpathogenic and circulated largely undetected for almost two decades until they rose to prominence following a series of outbreaks in the early 2000s. Despite lacking a functional toxin A, *C. difficile* RT 017 strains have been shown subsequently to be capable of causing disease as severe as that caused by strains producing both toxins A and B. While *C. difficile* RT 017 strains can be found in almost every continent today, epidemiological studies suggest that the RT is endemic in Asia and that the global spread of this MLST clade 4 lineage member is a relatively recent event. *C. difficile* RT 017 transmission appears to be mostly from human to human with only a handful of reports of isolations from animals. An important feature of *C. difficile* RT 017 strains is their resistance to several antimicrobials and this has been documented as a possible factor driving multiple outbreaks in different parts of the world. This review summarizes what is currently known regarding the emergence and evolution of strains belonging to *C. difficile* RT 017 as well as features that have allowed it to become an RT of global importance.

## Introduction

*Clostridium difficile* is an important cause of antimicrobial-associated diarrhoea (AAD) in both humans and animals [1]. In humans, the disease can progress from watery diarrhoea to life-threatening pseudomembranous colitis (PMC) and toxic megacolon [2]. *C. difficile* infection (CDI) is a toxin-mediated disease and major virulence factors include toxin A (TcdA, 308 kDa) and toxin B (TcdB, 270 kDa) [3]. An additional binary toxin (*C. difficile* transferase, CDT) is produced by some strains only. CDT-producing strains of *C. difficile* account for an increasing proportion of human infections in some parts of the world (currently ca. 20% of CDI cases in non-outbreak situations) but are common in animals [4,5]. *C. difficile* can be classified into different PCR ribotypes (RTs) using banding patterns of the amplified intergenic spacer region between the 16S and 23S rRNA genes [6]. Currently, over 600 RTs exist in the United Kingdom-based *C. difficile* Ribotyping Network (CDRN) database [7].

*C. difficile* RT 017 ranks among the most successful RTs of *C. difficile*. A toxigenic strain that produces only TcdB [8], RT 017 causes disease as severe as other toxigenic strains [9–12]. Although *C. difficile* RT 017 appears to have originated in Asia, it has spread globally and been responsible for multiple outbreaks around the world [13–23]. Few studies have been conducted to identify factors that may have contributed to the success of RT 017 [16,18]. This review summarizes what is known about *C. difficile* RT 017 regarding its history, characteristics, evolution, emergence and global dissemination.

## Brief history of *C. difficile* infection and the emergence of *C. difficile* RT 017

*C. difficile* (then named *Bacillus difficilis*) was first described in 1935 as part of neonatal gut flora. It produced a potent cytotoxin that caused tissue oedema, convolution and death when injected subcutaneously into guinea pigs and rabbits [24]. However, there were no reports of human gastrointestinal infections associated with *C. difficile* until 1978 when, after a period of intense trans-Atlantic competition between researchers, *C. difficile* was identified in faecal specimens from patients with PMC [25].

Not all strains of *C. difficile* produce toxins and cause disease. Initially, it was thought that all toxigenic strains of *C. difficile* produced both major toxins [26]. For two decades after the association between *C. difficile* and PMC was shown, it was believed that TcdA was required to cause initial damage to the intestinal mucosa before TcdB could exert its potent cytotoxic effect [27], and the significance of TcdA-negative, TcdB-positive (A-B+) stains was not apparent [17]. To further support this belief, the first few strains of *C. difficile* isolated with an A-B+ phenotype were associated only with asymptomatic carriage [28]. During this same period, there was a move away from using the faecal TcdB cytotoxicity assay and/or culture of *C. difficile* for diagnostic purposes due to the time and expense involved in maintaining and using cell lines, and the long turnaround time of culture. Concomitantly, there was an emphasis on developing rapid immunoassays for the detection of TcdA [29]. TcdA was chosen because of the continued mistaken belief that *C. difficile* produced either both TcdA and TcdB, or no toxins, because it was easier to manufacture antibodies against TcdA, and because detection of TcdA had greater sensitivity compared to detection of TcdB [30]. These tools made the detection of *C. difficile* easier, but with far less overall sensitivity, and further obscured the significance of A-B+ *C. difficile* strains.

The importance of A-B+ strains of *C. difficile* was finally appreciated at the end of the twentieth century when 16 patients in a Canadian tertiary-care hospital developed PMC with an A-B+ strain. Stool samples from these patients tested negative for *C. difficile* TcdA but were later shown via a cytotoxin assay to contain *C. difficile* that produced a functional TcdB only [17]. Similar findings were published from other countries [13,16] and further studies confirmed these strains as A-B+ *C. difficile* RT 017 [8]. At the same time, a study reported that not only could TcdB exert its cytotoxic effect in the absence of TcdA, but also that human intestinal mucosa was around 10 times more sensitive to TcdB than TcdA [31]. This was the first time that the clinical significance of A-B+ *C. difficile* became evident [32]. Over the last 20 years, *C. difficile* RT 017 has been isolated from many parts of the world, however, it is likely that *C. difficile* RT 017 originated from a single geographical region and its global dispersal has been a relatively recent event [33].

## Characteristics of *C. difficile* RT 017

### Epidemiological typing of *C. difficile* RT 017

Currently, PCR ribotyping is a method of typing *C. difficile* that is widely used in many parts of the world due to its relative simplicity and high discriminatory power [34]. However, ribotyping requires comparison of banding patterns with those of standard strains present in a library of patterns that was established in 1999 [6]. Thus, reports of *C. difficile* before or around that time classified *C. difficile* by various other methods [17,35]. Table 1 summarizes these different methods used when referring to *C. difficile* RT 017. Early ribotyping studies in Japan used their own nomenclature and assigned “fr” to RT 017 [36].

**Table 1.** *C. difficile* RT 017 categorized by other classification methods**.**

Classification Method	Type(s)	Reference
Serogrouping	F, X	[37]
Toxinotyping	VIII	[38]
NAP typing	NAP 9	[39]
REA grouping	CF1, CF2, CF3, CF4, CF5,CF6, CG1, CG3	[37]
MLST	ST 37, ST 45*	[40]
Ribotyping (internal nomenclature)	RT fr	[36]

Note: NAP; North American pulsed-field gel electrophoresis, REA; restriction endonuclease analysis, MLST; multilocus sequence typing, ST; sequence type, * a study in Thailand [41] performed MLST using a different database and classified RT 017 as ST 45.

Before genotype-based methods, *C. difficile* was classified using phenotypic methods that, in general, had poor reproducibility, low typeability, and lacked sufficient discriminatory power to be applied to epidemiological studies [42]. However, serogrouping was widely used early and showed a good correlation with toxigenicity [43]. Serogrouping classified *C. difficile* RT 017 as either serogroup F or X [37].

Many genotypic methods, including ribotyping, use unique banding patterns of different PCR products to classify *C. difficile* strains. Toxinotyping detects differences in the Pathogenicity Locus (PaLoc) and classifies *C. difficile* RT 017 as toxinotype VIII [38]. Pulsed-field gel electrophoresis is more commonly used in North America and classifies *C. difficile* RT 017 as North American pulsed-field gel electrophoresis type 9 (NAP 9) [39]. Restriction endonuclease analysis (REA) typing has greater discriminatory power than ribotyping and divides *C. difficile* RT 017 into several REA types which are grouped as REA groups CF and CG [37].

Multi-locus sequence typing (MLST) is another genotype-based method involving 7 housekeeping genes. However, it is not based on banding patterns but rather the unique sequences of these genes and thus has been used mainly in evolutionary studies. This method classifies *C. difficile* RT 017 as sequence type (ST) 37 belonging to evolutionary clade 4 [40]. MLST has good discriminatory power, however, it is relatively more complicated to perform [34]. The advent of next-generation sequencing makes *in silico* MLST now more accessible [44].

A recent study in China reported that RT 017 can also be identified using matrix-assisted laser desorption ionization time-of-flight mass spectrometry (MALDI-TOF MS) with high sensitivity and specificity [45]. However, this study did not include other *C. difficile* strains from clade 4 and another Chinese study suggested that different clade 4 strains may not be distinguishable by this method [46].

### *C. difficile* RT 017 toxin

**Figure 1.**
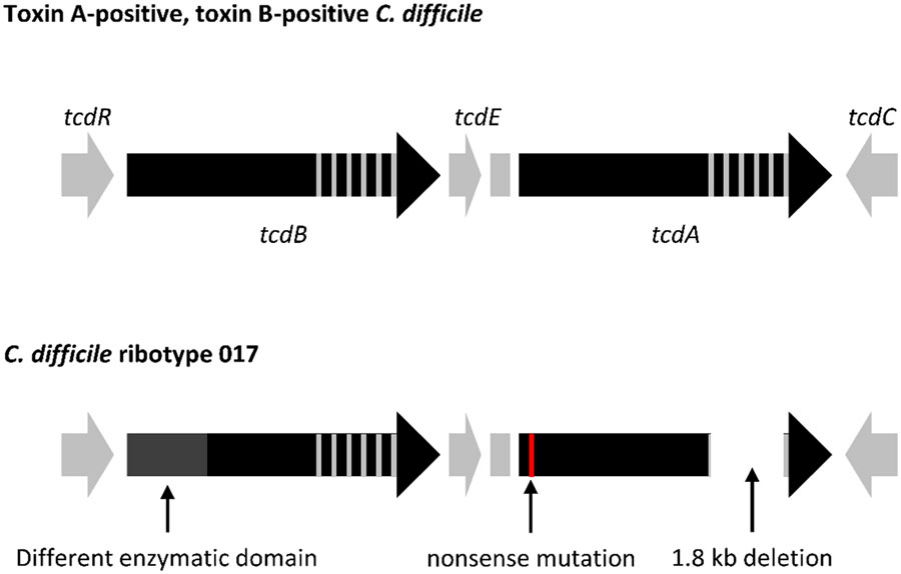
Comparative analysis of the PaLoc from C. difficile RT 017 and A + B + C. difficile strains. Arrows indicate open reading frames (ORFs) and the direction of transcription. The different enzymatic domain of the tcdB gene is responsible for the different CPE [48]. The nonsense mutation near the 5′ terminal of the tcdA gene is responsible for the loss of function of TcdA [49]. The 1.8 kb deletion near the 3′ terminal of the tcdA gene makes TcdA undetectable by many toxin EIAs [47].

*C. difficile* RT 017 is classified as A-B+ *C. difficile* as it produces only a functional TcdB [8]. Its TcdB also gives a different cytopathic effect (CPE) in cell cytotoxin assays using various cell lines compared to other strains that is often referred to as a variant CPE [16,47]. Studies on the *tcdA* gene of *C. difficile* RT 017 revealed a 1.8 kb deletion in the repeating region (3′ end) (Figure 1) and a point mutation in the 5′ end which results in a premature stop codon [49,50]. The 1.8 kb deletion corresponds to a deletion of the carboxy repetitive oligopeptide (CROP) region of TcdA, which is the recognition site of many TcdA enzyme immunoassays (EIAs), making the toxin undetectable by these EIAs [47]. The nonsense mutation at 5′ end corresponds with a loss of catalytic action of the TcdA, thus making the toxin non-functional [47,49].

Notably, despite lacking a functional TcdA, most of the *tcdA* gene in *C. difficile* RT 017 remains intact and can be detected by PCR if primers specific to the non-repeating region of the *tcdA* gene are used. In such cases, *C. difficile* RT 017 could be incorrectly detected as both *tcdA*- and *tcdB*-positive *C. difficile* [51]. While these primers are efficient for detection of toxigenic strains in clinical practice, the results may appear confusing in an epidemiological study. An additional primer set is needed to identify the deletion in the repeating region of *tcdA* gene and differentiate *C. difficile* RT 017 from true A+B+ *C. difficile* strains [28,52].

**Figure 2.**
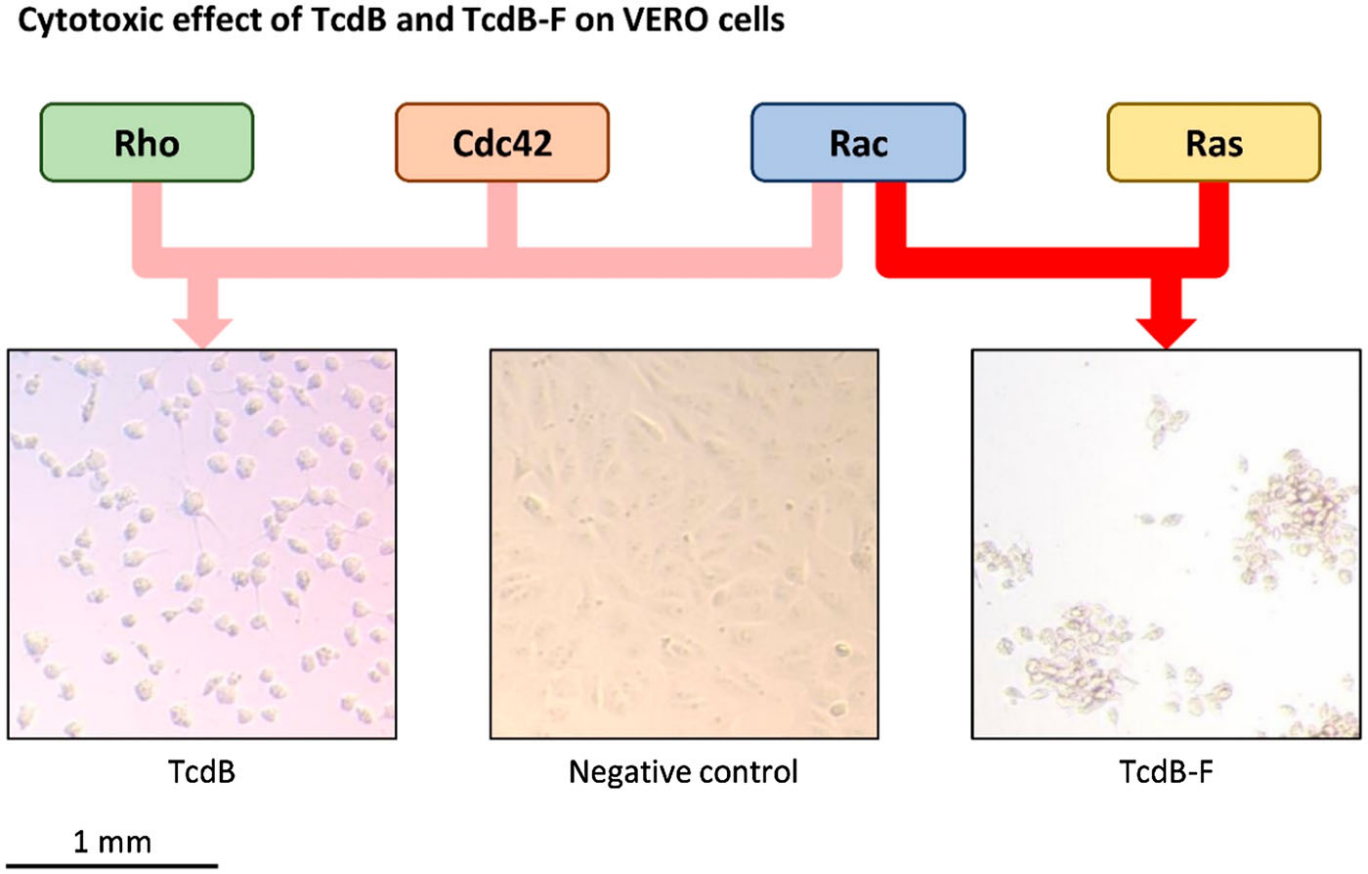
The cytotoxic effect of TcdB and TcdB-F on VERO cells. VERO cells were treated with the supernatant of 72-hour-old cultures of C. difficile strain 2149 (RT 014/020 which produces TcdB), C. difficile strain 1470 (RT 017 which produce TcdB-F), and C. difficile ATCC 700057 (RT 038 which is non-toxigenic) and incubated at 37°C for 24 hours before inspection under a light microscope. TcdB glycosylates Rho, Rac, and Cdc42 targets resulting in arborization of cells while TcdB-F glycosylates Rac and Ras targets resulting in rounding of cells without arborization.

Interestingly, the TcdB of RT 017 (TcdB-F) is different from the TcdB commonly found in most *C. difficile* strains. TcdB-F behaves as a “functional hybrid,” combining characteristics of both TcdB and the *Clostridium sordellii* lethal toxin, TclS. While TcdB-F binds to the same cellular receptors as TcdB, the two proteins display differences in their target specificity, with TcdB primarily glucosylating Rho, Rac and Cdc42 targets, and TcdB-F glucosylating Rac and Ras targets (Figure 2) [48]. The difference in cellular targets is thought to be responsible for the different CPE observed for the two toxins [50].

### Infection due to *C. difficile* RT 017

Despite producing toxin B only, several studies suggest that *C. difficile* RT 017 causes clinical disease that is indistinguishable from that caused by other *C. difficile* RTs [9,12]. In addition, *C. difficile* RT 017 causes disease as severe as that caused by “hypervirulent” *C. difficile* RT 027 [10]. In an outbreak setting, mortality due to *C. difficile* RT 017 can be as high as 37.5% [47], but this high mortality rate may have been due to the exclusion of mild cases. There have been no clinical studies of *C. difficile* RT 017 infection in South East Asia, where there is a high prevalence of RT 017 [41,53,54]. Given that CDI in this region was, in general, associated with low mortality and recurrence [55], it will be interesting to see whether the less severe CDI in this region is specifically associated with *C. difficile* RT 017 or if there are other unknown protective factors in the population or region, such as a high prevalence of carriage of non-toxigenic strains, which may occupy the same niche and competitively exclude toxigenic strains from the gut [53,56,57].

## Evolution and transmission of *C. difficile* RT 017

**Figure 3.**
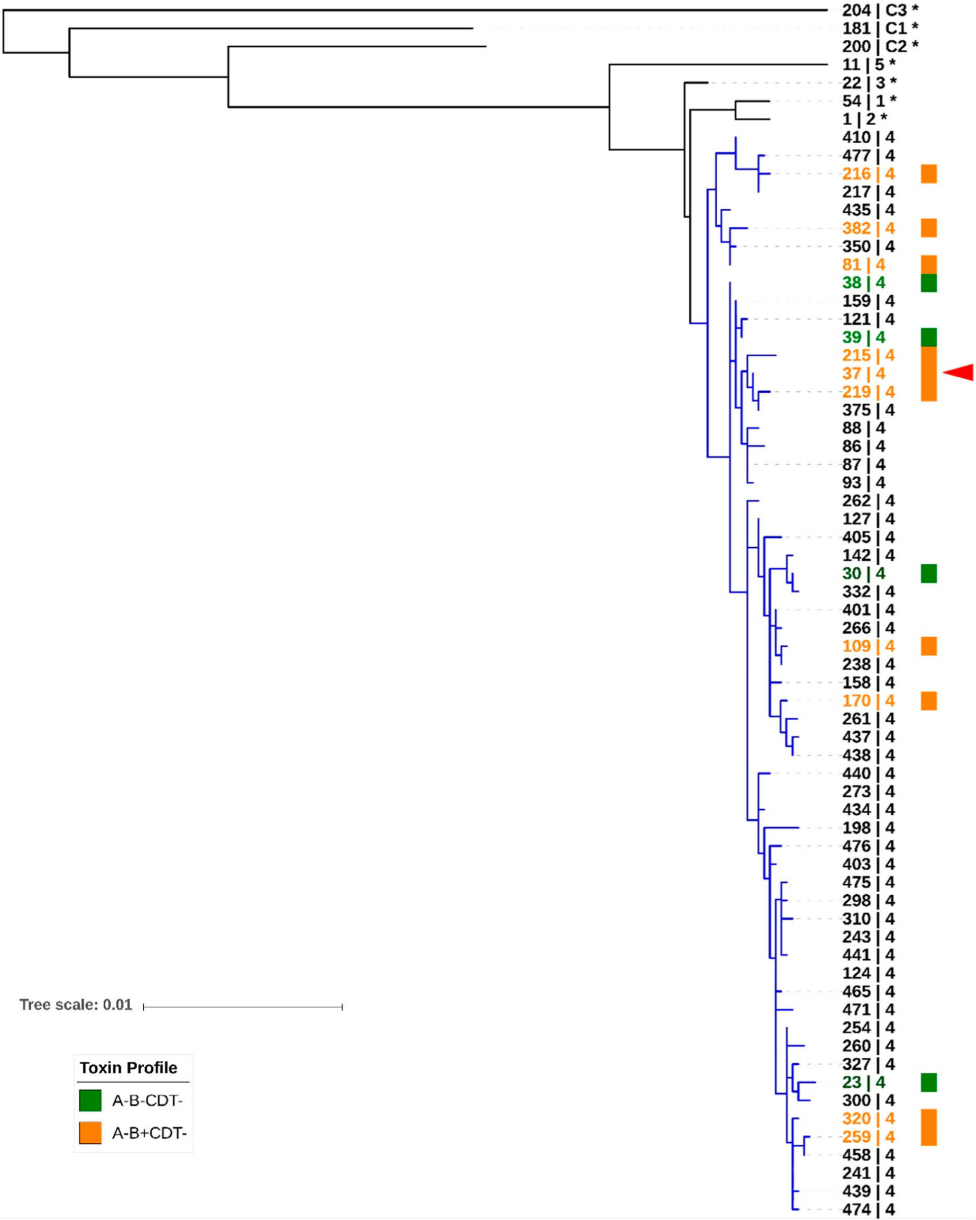
Sequence type diversity in evolutionary clade 4. Maximum-likelihood MLST phylogeny. Sequences were aligned using MUSCLE and tree was generated in MEGA7 with evolutionary distances calculated using the Tajima-Nei model. The scale shows the number of nucleotide substitution per site, based on concatenated MLST allele sequences (7 loci, 3501 bp). The tree is mid-point rooted and supported by 500 bootstrap replicates (only values >50 are shown). For global phylogenetic context, well-characterised representatives of MLST clade 1 (ST 54), 2 (ST 1), 3 (ST 22), 5 (ST 11), C1 (ST 181), C2 (ST 200), and C3 (ST 204) are also shown (*). Branches for clade 4 are shown in blue. Known toxin profiles of clade 4 strains are indicated by orange (A-B+CDT-) and green (A-B-CDT-) colour. RT 017 (ST 37) is indicated with a red arrowhead.

Based on MLST and Bayesian evolutionary model analysis (Figure 3), *C. difficile* has evolved into at least five clades and three cryptic clades. This clade divergence occurred more than a million years ago [34]. *C. difficile* RT 017 (ST 37; red arrowhead in Figure 3) is a member of *C. difficile* clade 4 along with many non-toxigenic, and some similar toxigenic, strains [46,58–61]. Despite limited data, it is clear that both A-B+CDT- and non-toxigenic strains of *C. difficile* (orange and green, respectively, in Figure 3) are equally distributed throughout clade 4, indicating that the clade 4 ancestor could either be a toxigenic (A-B+CDT-) or non-toxigenic strain. A recent study analyzed time-scaled core-genome phylogenies and suggested that the clade 4 ancestor was a non-toxigenic strain of *C. difficile*, and that acquisition of the PaLoc in *C. difficile* RT 017 occurred around 500 years ago [59].

**Figure 4.**
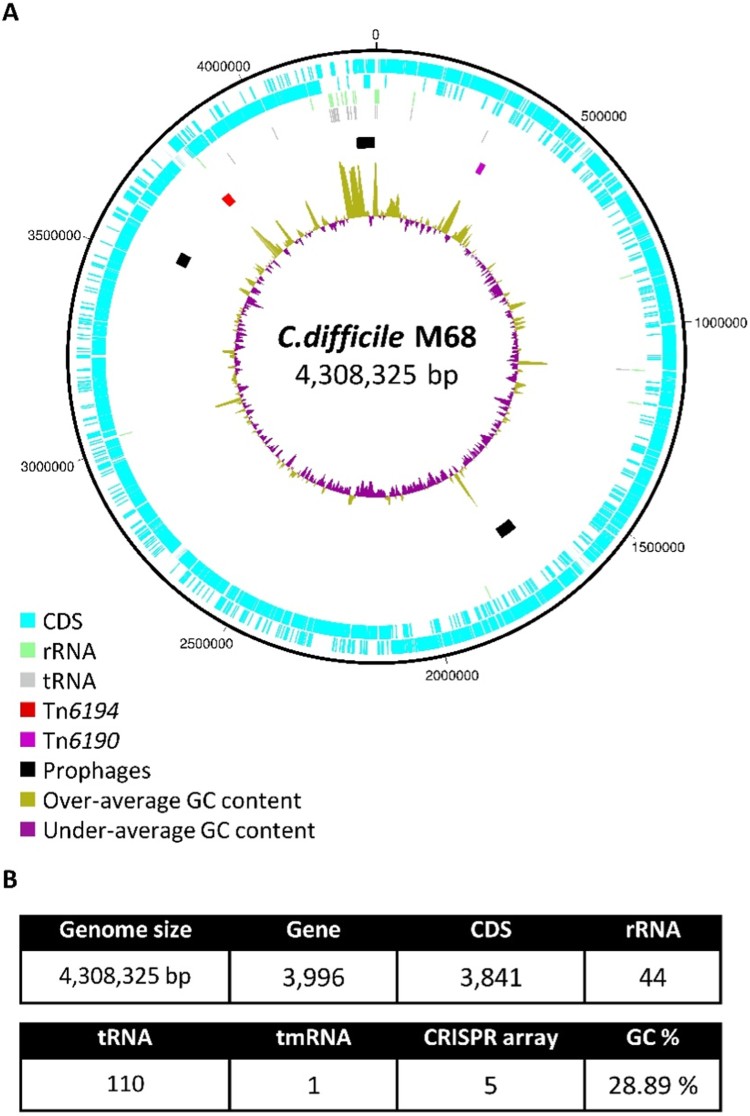
A. Circular representation of the genome of C. difficile strain M68 (RT 017, ST 37, GenBank accession number NC017175.1). From outside to inside, the concentric circles represent (1) and (2) all coding sequences (CDS) transcribed in clockwise and counter-clockwise, (3) all rRNA, (4) all tRNA, (5) transposons (Tn6194 containing ermB gene represented in red and Tn6190 containing tetM gene represented in purple) and prophages (counterclockwise from top; ΦCDHM19 [58,163 bp, GC% = 31.34%], ΦCDHM13 [39,325 bp, GC% = 29.34%], and ΦMMP01 [55,106 bp, GC% = 28.87%]), and (6) GC content. B. Key characteristics of the genome.

To date, the genomes of two *C. difficile* RT 017 strains (CF5, isolated in Belgium in 1995, and M68, isolated in Ireland in 2006) have been completely sequenced, providing important reference chromosomes for whole genome sequencing (WGS) studies of this lineage [62]. Figure 4 shows the genome of *C. difficile* strain M68. Using WGS, Cairns *et al*. showed that 23 of 24 of *C. difficile* RT 017 strains from one hospital were closely related and formed a single cluster. The only unrelated *C. difficile* RT 017 strain was isolated from a patient with community-acquired CDI and this belonged to a cluster from outer London hospitals. These findings suggested that *C. difficile* RT 017 was mostly transmitted between patients in the same ward and between wards in the same hospital. The study further found that environmental contamination with clinical isolates was possible and that RT 017 could withstand decontamination with hydrogen peroxide vapour [22].

Another WGS study of 277 different *C. difficile* RT 017 strains isolated from around the world, including 24 from animals (cattle, dogs, and horses) showed that *C. difficile* RT 017 could be transmitted between humans and animals, and also reported that deletions and insertions found in RT 017 genomes were distributed throughout all geographical areas [33]. The finding of little genetic diversity implies that *C. difficile* RT 017 originated in a single geographical area and that global spread occurred relatively recently, however, it remained unclear where that single geographical area was. Cairns *et al*. [33] concluded that *C. difficile* RT 017 originated in North America and then spread to Europe, Asia and other parts of the world [33]. This conclusion contradicts many epidemiological studies (see below) that, taken collectively, suggest that the origin of *C. difficile* RT 017 is in Asia. The Cairns *et al.* study included only a limited number of historic *C. difficile* RT 017 isolates from Asia (2 strains from Korea and 1 strain from Japan, all isolated in 1995) and a greater number of *C. difficile* RT 017 strains from North America (9 strains from the United States isolated from 1990 to 1996).

## Global dissemination of *C. difficile* RT 017

**Figure 5.**
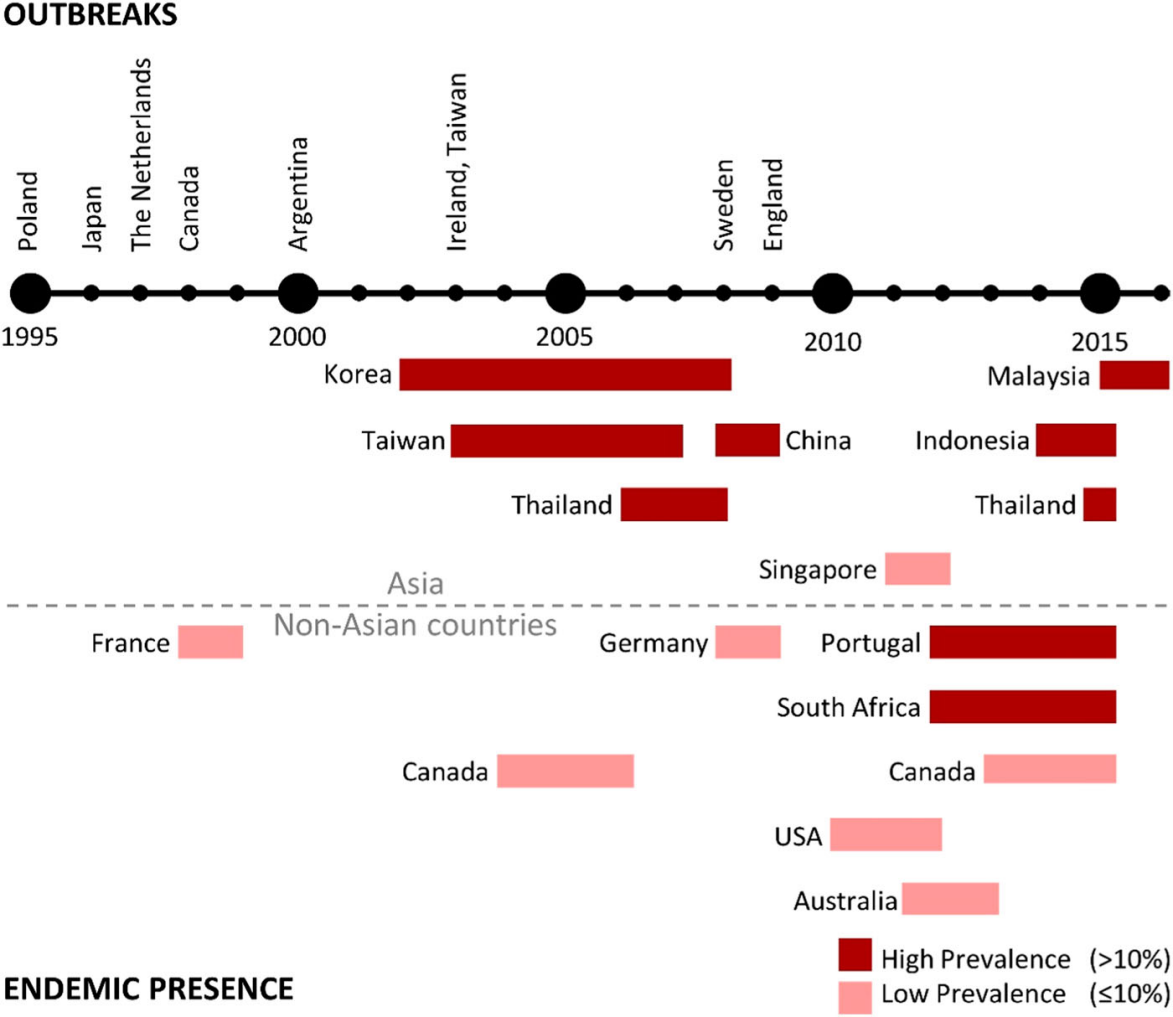
Timeline of *C. difficile* RT 017 reports around the world. Outbreaks refer to an increase in the regional prevalence of RT 017, which is confirmed either to be clonal or with evidence suggesting that isolates came from the same source. Endemic presence refers to prevalence reports that were not associated with outbreaks.

Despite producing only one toxin, *C. difficile* RT 017 has successfully spread throughout the world with evidence of human infection in North America [17,39,47,63–66], Europe [8,13,16,20,22,23,67,68], Asia [9,14,15,19,69–76], South America [18], Africa [77], and Australia [78–81]. Figure 5 summarizes chronologically the major events surrounding the detection of *C. difficile* RT 017 from around the world, comparing studies of prevalence during outbreaks to studies in non-outbreak settings.

Reports on *C. difficile* RT 017 infection started in the late 1990s with a series of outbreaks in Poland [13], Japan [14,15], the Netherlands [16], Canada [17], and Argentina [18]. During the early 2000s, there were outbreaks of so-called “hypervirulent” *C. difficile* RT 027 in Europe and North America [82], and the number of RT 017 reports appeared to subside [39,63,67,68,83–85]. Still, there were further outbreaks of RT 017 infection in Ireland and Taiwan during 2003 and 2004 [19–21], and in Sweden in 2008 [23]. The most recent documented outbreak of RT 017 infection started in 2009 in England and persisted for at least 3 years [22].

Among these *C. difficile* RT 017 outbreaks, clinical characteristics of the patients were described only in reports from the outbreak in Canada, with 18.8% (3/16) of cases having PMC, 31.3% (5/16) of cases being recurrent and a 37.5% (6/16) mortality rate [17,47]. Outbreaks of *C. difficile* RT 017 infection have been linked to the use of clindamycin [16] and fluoroquinolones [21]. In both outbreaks, discontinuation of the offending agent resulted in a rapid decline in the number of CDI cases due to *C. difficile* RT 017 [16,21]. This suggests that these outbreaks were associated with the use of specific antimicrobials and that antimicrobial stewardship helped to control spread.

Besides many outbreaks, there have also been non-outbreak reports of *C. difficile* RT 017 throughout the world. The majority of these reports with high prevalence figures were from Asia, while reports from non-Asian countries mostly recorded low prevalence figures. Data summarizing the prevalence of *C. difficile* RT 017 in Asia and non-Asian countries can be found in Tables S1 and S2 in the supplementary document.

### *C. difficile* RT 017 in Asia

It is likely that *C. difficile* RT 017 is endemic in Asia and has been resident in this region for a long time, for three different reasons. First, in contrast to non-Asian countries, RT 017 appeared mainly in non-outbreak-related prevalence studies [41,53,54,69,71–75,86–88]. Second, there have been reports of A-B+CDT- *C. difficile* RTs in the region other than *C. difficile* RT 017 with similar deletions in the *tcdA* gene, some of which have also been classified in MLST clade 4 [46,58,60,61,71,89]. Third, the earliest Asian isolates of RT 017 in humans can be dated back to 1993 in Indonesia, where five strains of RT 017 were isolated from healthy infants [15]. The high prevalence and diversity of A-B+CDT- *C. difficile* in Asia and the evidence of old *C. difficile* RT 017 isolates suggest that the origin of this RT is in Asia. While Asia is a very large continent, current information suggests that *C. difficile* RT 017 is endemic in at least two different regions of the continent: parts of East Asia, and South East Asia [90].

#### East Asia

East Asia can be geographically divided into Japan and the mainland section which consists of China (including Hong Kong), North and South Korea, and the island of Taiwan. The prevalence of different *C. difficile* RTs in these two areas varies with RT 017 being a predominant strain only in the mainland section plus Taiwan [9,19,69–75]. Historically, RT 017 has been responsible for ca. 15–40% of patients with CDI in South Korea [9,69–71], China [72–74], and Taiwan [19,75]. In Taiwan, there was an increase in the prevalence of *C. difficile* RT 017 that resembled an outbreak in 2004 (73.3%; 11/15), but the prevalence eventually decreased to an endemic rate of 23.9% (11/46) in 2007 [19].

In contrast to these reports, Japan saw an outbreak of *C. difficile* RT 017 infection in 1996 [14,15], perhaps coincidentally, around the same time as RT 017 outbreaks in Poland, the Netherlands and Canada [13,16,17,67]. However, there have been no major reports of *C. difficile* RT 017 infection in Japan since. Interestingly, in 2001, there was an outbreak of CDI caused by an A-B+ strain of *C. difficile* with an RT pattern resembled *C. difficile* RT 017 [91]. This strain was later identified as the novel *C. difficile* RT 369, a strain that is closely related to *C. difficile* RT 017 [36], and that was recently identified in China as ST 81, a single loci variant of ST 37 [92]. To date, RT 369 remains among the most common toxigenic strains isolated in Japan while only a small number of *C. difficile* strains belonging to RT 017 have been detected [93].

#### South East Asia

Most epidemiological studies in South East Asia have been conducted in Thailand [41,53,89] with additional reports from Indonesia [54], Laos [86], Malaysia [56,87] and Singapore [88]. Although the information is limited, based on these publications, and some publications from Thailand that detected a high prevalence of A-B+ *C. difficile* [94–96], it is likely that RT 017 is endemic throughout this region.

Despite isolating *C. difficile* RT 017 strains as early as 1993 [15], there were no epidemiological studies in the region until 2006 [41]. All studies thereafter reported similar results. In Thailand, three studies confirmed that *C. difficile* RT 017 ranks among the most common toxigenic strains present (ca. 30.8% – 41.5%) [41,53,89]. In Indonesia, *C. difficile* RT 017 was the most prevalent RT isolated from patients [54]. *C. difficile* RT 017 has been isolated in Laos [86], although only five patients were included in this report. The most recent report from South East Asia came from Malaysia where the prevalence of *C. difficile* RT 017 was 20.0% [56]. In contrast to other South East Asian countries, a study in Singapore reported a low prevalence of RT 017 of 4.9% (3/61), and an RT distribution more like European countries. The comment was made that this possibly reflected the international population of Singapore, both resident and passing through [88].

### *C. difficile* RT 017 in non-Asian countries

Outside Asia, *C. difficile* RT 017 is mostly associated with outbreaks. The first group of outbreaks was reported from 1995 to 1998 in Poland [13], the Netherlands [16] and Canada [17]. These outbreaks occurred during the same time-frame as the Japanese outbreak [14,15]. Since 2000, there have been four outbreaks of *C. difficile* RT 017 infection outside Asia [18,20–23]. Even though there have been non-outbreak reports of RT 017 in some parts of the world, the prevalence is low in most areas (≤10%) when compared to Asia [8,13,16,20,22,39,63,67,68,78–81].

#### North America

After 2002, *C. difficile* RT 017 was rapidly overshadowed by the emergence of the “hyper-virulent” *C. difficile* RT 027 in this region [82]. The prevalence of *C. difficile* RT 017 in Canada decreased from 5.4% (58/1,080) during 2004–2006 [63] to 1.3% (17/1,310) during 2013–2015 [83]. The prevalence of *C. difficile* RT 017 in the United States was ca. 2–3% during 2010–2012 [64–66]. In 2011, the overall prevalence of RT 017 in North America was reported at 4.3% (15/350) of toxigenic strains [39].

#### Europe

Apart from obvious outbreaks, reports of RT 017 in Europe were scarce. During the late 1990s, the prevalence of RT 017 was 2.5% (9/364) in France [67]. During 2008–2009, RT 017 was responsible for 4.9% (2/41) of severe CDI cases in Germany [84]. In 2012, only one out of 171 (0.6%) *C. difficile* isolates from Austria was classified as *C. difficile* RT 017 [68]. A pan-European study reported an overall prevalence of *C. difficile* RT 017 during 2011–2014 of 1.8% (16/866) [85]. Portugal was the only European country to report a prevalence of *C. difficile* RT 017 higher than 10% [97].

#### Australia

Several epidemiological studies conducted in various regions of Australia with *C. difficile* RT 017 being found at a much lower prevalence compared to Asia. The prevalence of *C. difficile* RT 017 infection was ca. 3% [78–81] suggesting that those cases are are more likely to be imported rather than caused by endemic strains.

#### Africa

The number of studies on CDI in Africa is very limited. To date, the only country with reported *C. difficile* RT 017 infection is South Africa, where a very high prevalence of RT 017 among diarrhoeal patients in tuberculosis hospitals was seen [77,98,99]. Historically, Cape Town in South Africa has been an important port city where ships coming from and going to Asia, Australia and Europe stopped during their voyages. The introduction of *C. difficile* RT 017 may merely reflect travel between these regions, however, it appears that *C. difficile* RT 017 has now become established within the hospital system in South Africa. Patients testing positive for *C. difficile* are at high risk of mortality, and tuberculosis is an additional risk factor for CDI in populations with HIV [100].

### *C. difficile* RT 017 in animals

Recently, many *C. difficile* strains associated with CDI in humans have also been isolated from animals or animal products suggesting that CDI may be transmitted from animals [101]. Despite its high prevalence in the Asian human population [102], there have never been any reports of *C. difficile* RT 017 in animals in this region [103,104], and it has rarely been reported in animals elsewhere. *C. difficile* RT 017 has been isolated from calves in Canada [105] and rabbits in Italy [106]. The WGS study undertaken in the United Kingdom by Cairns *et al*. involving 277 *C. difficile* RT 017 strains only included 24 strains of animal origin [33]. The reasons why RT 017 is apparently not prevalent in animals have not been elucidated.

## Role of antimicrobial resistance in the outbreaks of *C. difficile* RT 017

AMR plays an important role in the dissemination of many *C. difficile* RTs. Being resistant to antimicrobials while the intestinal microbiota is disrupted allows *C. difficile* to survive, produce toxins and eventually cause disease [2]. Furthermore, being intrinsically resistant to alcohol and desiccation, *C. difficile* as a spore can survive within the hospital environment and spread to patients. Antimicrobial resistance has been associated with CDI outbreaks in the past; in particular, the outbreaks of “epidemic” *C. difficile* RT 027 in North America and Europe were associated with fluoroquinolone and rifampicin resistance.

Outbreaks of infection with *C. difficile* RT 017 have been linked with clindamycin- and fluoroquinolone-resistant strains [13,16,18,21]. Besides these antimicrobials, *C. difficile* RT 017 also has higher rates of resistance to tetracyclines and rifaximin [107–109]. Tetracycline resistance was associated with an outbreak of *C. difficile* RT 078 [110,111]. Rifaximin is a derivative of rifampicin which was also associated with the outbreak of *C. difficile* RT 027 [112,113]. There is no doubt that misuse of these antimicrobials may lead to the future outbreaks of *C. difficile* RT 017, given that it is endemic in East and South East Asia, where tetracycline and rifampicin are commonly prescribed for many tropical infections and tuberculosis, respectively.

## Conclusions

*C. difficile* RT 017 is one of the most successful RTs of *C. difficile* in the world. It was the first A-B+ *C. difficile* shown to cause CDI following several outbreaks. This discovery led to a better understanding of the pathogenesis of CDI in general, together with the roles of TcdA and TcdB, and eventually lead to changes in the way the laboratory diagnosis of CDI was made. The high rate of resistance to many antimicrobial agents provides hints as to how *C. difficile* RT 017 spread throughout the globe. It also gives us a warning that antimicrobial stewardship is needed to prevent further outbreaks.

The ancestral home of *C. difficile* RT 017 remains controversial, however, the weight of epidemiological evidence suggests that this strain originated in Asia and spread to other regions of the world long before the much-publicised spread of RT 027. Particular clinical characteristics of *C. difficile* RT 017 infection have yet to be determined. Why *C. difficile* RT 017 is not found more commonly in animals despite successful human spread also remains unclear, however, this may just reflect a lack of animal studies in Asia. Also, there has been no study comparing phenotypic characteristics of *C. difficile* RT 017, such as sporulation, germination and motility, with other epidemic strains. Since these properties are related to the spread of *C. difficile*, such studies may uncover important factors that help in the control of *C. difficile* RT 017 spread and prevent further outbreaks.

## Supplementary Material

Supplemental Material
