# Supplementary material for: Clostridium difficile ribotype 017 – characterization, evolution and epidemiology of the dominant strain in Asia
Source: Emerg Microbes Infect. 2019 May 29;8(1):796–807. doi: 10.1080/22221751.2019.1621670 (PMC6542179; doi:10.1080/22221751.2019.1621670)
Supplement: Supplemental Material [file TEMI_A_1621670_SM1223.docx]

**Table S1 – Prevalence of C. difficile RT 017 in non-outbreak settings in Asia**

| **Country** | **Year** | **Number of Strains** | **Prevalence** | **Reference** |
| --- | --- | --- | --- | --- |
| **East Asia**  South Korea  China  Taiwan  **Southeast Asia**  Thailand  Indonesia  Malaysia  Laos  Singapore | 1980 – 2006  2006 – 2008  2009 – 2010  2010 – 2013  N/A  2008 – 2009  2009 – 2013  2012 – 2015  2002 – 2007  2011 – 2013  2006 – 2008  2015  2014 – 2015  2015 – 2016  2013  2011 – 2012 | 462  408  140  510  75  110 *  405 *  411  110 *  120 *  53 *  105  74  100  5  61 | 21.4%  25.7%  15.7%  27.3%  33.3%  37.3%  13.6%  16.5%  36.4%  43.3%  41.5%  11.4%  24.3%  20.0%  20.0%  4.9% | [1]  [2]  [3]  [4]  [5]  [6]  [7]  [8]  [9]  [10]  [11]  [12]  [13]  [14]  [15]  [16] |

(Note: * only toxigenic *C. difficile* strains were included in these studies.)

**Table S2 – Prevalence of C. difficile RT 017 in non-outbreak settings in non-Asian countries**

| **Country** | **Year** | **Number of Strains** | **Prevalence** | **Reference** |
| --- | --- | --- | --- | --- |
| **North America**  Canada  The United States  Entire North America | 2004 – 2006  2013 – 2015  2010 – 2012  2011  2011  2011 | 1080  1310  33  1364  720  350 | 5.4%  1.3%  1.9%  2.3%  2.8%  4.3% | [17]  [18]  [19]  [20]  [21]  [22] |
| **Europe**  France  Germany  Portugal | 1998 – 1999  2008 – 2009  2012 - 2015 | 364  41 *  191 | 2.5%  4.9%  13.1% | [23]  [24]  [25] |
| **Australia**  Australia | 2010 – 2012  2012  2013 – 2014 | 657  542  151 | 2.9%  3.3%  2.0% | [26]  [27]  [28] |
| **Africa**  South Africa | 2012 – 2103  2014 – 2015 | 32  269 | 50.0%  64.7% | [29]  [30] |

(Note: All studies only included toxigenic *C. difficile* strains; * only toxigenic *C. difficile* strains from patients with severe CDI were included in this study.)

# References

1. Kim H, Riley TV, Kim M, et al. Increasing prevalence of toxin A-negative, toxin B-positive isolates of *Clostridium difficile* in Korea: impact on laboratory diagnosis. J Clin Microbiol. 2008 Mar;46(3):1116-7. doi: 10.1128/JCM.01188-07. PubMed PMID: 18199783; PubMed Central PMCID: PMCPMC2268382.

2. Kim H, Jeong SH, Roh KH, et al. Investigation of toxin gene diversity, molecular epidemiology, and antimicrobial resistance of *Clostridium difficile* isolated from 12 hospitals in South Korea. Korean J Lab Med. 2010 Oct;30(5):491-7. doi: 10.3343/kjlm.2010.30.5.491. PubMed PMID: 20890081.

3. Kim J, Kang JO, Kim H, et al. Epidemiology of *Clostridium difficile* infections in a tertiary-care hospital in Korea. Clin Microbiol Infect. 2013 Jun;19(6):521-7. doi: 10.1111/j.1469-0691.2012.03910.x. PubMed PMID: 22712697.

4. Kim J, Kim Y, Pai H. Clinical characteristics and treatment outcomes of *Clostridium difficile* infections by PCR ribotype 017 and 018 strains. PLoS One. 2016;11(12):e0168849. doi: 10.1371/journal.pone.0168849. PubMed PMID: 28002482; PubMed Central PMCID: PMCPMC5176314.

5. Huang H, Fang H, Weintraub A, et al. Distinct ribotypes and rates of antimicrobial drug resistance in *Clostridium difficile* from Shanghai and Stockholm. Clin Microbiol Infect. 2009 Dec;15(12):1170-3. doi: 10.1111/j.1469-0691.2009.02992.x. PubMed PMID: 19624517.

6. Huang H, Weintraub A, Fang H, et al. Antimicrobial susceptibility and heteroresistance in Chinese *Clostridium difficile* strains. Anaerobe. 2010 Dec;16(6):633-5. doi: 10.1016/j.anaerobe.2010.09.002. PubMed PMID: 20849968.

7. Chen YB, Gu SL, Shen P, et al. Molecular epidemiology and antimicrobial susceptibility of *Clostridium difficile* isolated from hospitals during a 4-year period in China. J Med Microbiol. 2018 Jan;67(1):52-59. doi: 10.1099/jmm.0.000646. PubMed PMID: 29160203.

8. Jin D, Luo Y, Huang C, et al. Molecular epidemiology of *Clostridium difficile* infection in hospitalized patients in Eastern China. J Clin Microbiol. 2017 Mar;55(3):801-810. doi: 10.1128/JCM.01898-16. PubMed PMID: 27974547; PubMed Central PMCID: PMCPMC5328448.

9. Chia JH, Lai HC, Su LH, et al. Molecular epidemiology of *Clostridium difficile* at a medical center in Taiwan: persistence of genetically clustering of A(-)B(+) isolates and increase of A(+)B(+) isolates. PLoS One. 2013;8(10):e75471. doi: 10.1371/journal.pone.0075471. PubMed PMID: 24116048; PubMed Central PMCID: PMCPMC3792110.

10. Hung YP, Huang IH, Lin HJ, et al. Predominance of *Clostridium difficile* ribotypes 017 and 078 among toxigenic clinical isolates in Southern Taiwan. PLoS One. 2016;11(11):e0166159. doi: 10.1371/journal.pone.0166159. PubMed PMID: 27861606; PubMed Central PMCID: PMCPMC5115699.

11. Ngamskulrungroj P, Sanmee S, Putsathit P, et al. Molecular epidemiology of *Clostridium difficile* infection in a large teaching hospital in Thailand. PLoS One. 2015;10(5):e0127026. doi: 10.1371/journal.pone.0127026. PubMed PMID: 26000789; PubMed Central PMCID: PMCPMC4441498.

12. Putsathit P, Maneerattanaporn M, Piewngam P, et al. Prevalence and molecular epidemiology of *Clostridium difficile* infection in Thailand. New Microbes New Infect. 2017 Jan;15:27-32. doi: 10.1016/j.nmni.2016.10.004. PubMed PMID: 28119780; PubMed Central PMCID: PMCPMC5237757.

13. Collins DA, Gasem MH, Habibie TH, et al. Prevalence and molecular epidemiology of *Clostridium difficile* infection in Indonesia. New Microbes New Infect. 2017 Jul;18:34-37. doi: 10.1016/j.nmni.2017.04.006. PubMed PMID: 28603640; PubMed Central PMCID: PMCPMC5453861.

14. Riley TV, Collins DA, Karunakaran R, et al. High prevalence of toxigenic and non-toxigenic *Clostridium difficile* in Malaysia. J Clin Microbiol. 2018 Mar 21;56(6):e00170-18. doi: 10.1128/JCM.00170-18. PubMed PMID: 29563206.

15. Cheong E, Roberts T, Rattanavong S, et al. *Clostridium difficile* infection in the Lao People's Democratic Republic: first isolation and review of the literature. BMC Infect Dis. 2017 Sep 21;17(1):635. doi: 10.1186/s12879-017-2737-6. PubMed PMID: 28934954; PubMed Central PMCID: PMCPMC5609038.

16. Tan XQ, Verrall AJ, Jureen R, et al. The emergence of community-onset *Clostridium difficile* infection in a tertiary hospital in Singapore: a cause for concern. Int J Antimicrob Agents. 2014 Jan;43(1):47-51. doi: 10.1016/j.ijantimicag.2013.09.011. PubMed PMID: 24290727.

17. Martin H, Willey B, Low DE, et al. Characterization of *Clostridium difficile* strains isolated from patients in Ontario, Canada, from 2004 to 2006. J Clin Microbiol. 2008 Sep;46(9):2999-3004. doi: 10.1128/JCM.02437-07. PubMed PMID: 18650360; PubMed Central PMCID: PMCPMC2546775.

18. Karlowsky JA, Adam HJ, Kosowan T, et al. PCR ribotyping and antimicrobial susceptibility testing of isolates of *Clostridium difficile* cultured from toxin-positive diarrheal stools of patients receiving medical care in Canadian hospitals: the Canadian *Clostridium difficile* Surveillance Study (CAN-DIFF) 2013-2015. Diagn Microbiol Infect Dis. 2018 Jan 31;91(2):105-111. doi: 10.1016/j.diagmicrobio.2018.01.017. PubMed PMID: 29456070.

19. Dubberke ER, Reske KA, Seiler S, et al. Risk factors for acquisition and loss of *Clostridium difficile* colonization in hospitalized patients. Antimicrob Agents Chemother. 2015 Aug;59(8):4533-43. doi: 10.1128/AAC.00642-15. PubMed PMID: 25987626; PubMed Central PMCID: PMCPMC4505269.

20. Lessa FC, Mu Y, Bamberg WM, et al. Burden of *Clostridium difficile* infection in the United States. N Engl J Med. 2015 Jun 11;372(24):2369-70. doi: 10.1056/NEJMc1505190. PubMed PMID: 26061850.

21. Waslawski S, Lo ES, Ewing SA, et al. *Clostridium difficile* ribotype diversity at six health care institutions in the United States. J Clin Microbiol. 2013 Jun;51(6):1938-41. doi: 10.1128/JCM.00056-13. PubMed PMID: 23554188; PubMed Central PMCID: PMCPMC3716112.

22. Tenover FC, Akerlund T, Gerding DN, et al. Comparison of strain typing results for *Clostridium difficile* isolates from North America. J Clin Microbiol. 2011 May;49(5):1831-7. doi: 10.1128/JCM.02446-10. PubMed PMID: 21389155; PubMed Central PMCID: PMCPMC3122689.

23. Barbut F, Lalande V, Burghoffer B, et al. Prevalence and genetic characterization of toxin A variant strains of *Clostridium difficile* among adults and children with diarrhea in France. J Clin Microbiol. 2002 Jun;40(6):2079-83. PubMed PMID: 12037068; PubMed Central PMCID: PMCPMC130789.

24. Arvand M, Hauri AM, Zaiss NH, et al. *Clostridium difficile* ribotypes 001, 017, and 027 are associated with lethal *C. difficile* infection in Hesse, Germany. Euro Surveill. 2009 Nov 12;14(45). PubMed PMID: 19941785.

25. Isidro J, Santos A, Nunes A, et al. Imipenem resistance in *Clostridium difficile* ribotype 017, Portugal. Emerg Infect Dis. 2018 Apr;24(4):741-745. doi: 10.3201/eid2404.170095. PubMed PMID: 29553322; PubMed Central PMCID: PMCPMC5875251.

26. Eyre DW, Tracey L, Elliott B, et al. Emergence and spread of predominantly community-onset *Clostridium difficile* PCR ribotype 244 infection in Australia, 2010 to 2012. Euro Surveill. 2015 Mar 12;20(10):21059. PubMed PMID: 25788254.

27. Collins DA, Putsathit P, Elliott B, et al. Laboratory-based surveillance of *Clostridium difficile* strains circulating in the Australian healthcare setting in 2012. Pathology. 2017 Apr;49(3):309-313. doi: 10.1016/j.pathol.2016.10.013. PubMed PMID: 28237369.

28. Knight DR, Giglio S, Huntington PG, et al. Surveillance for antimicrobial resistance in Australian isolates of *Clostridium difficile*, 2013-14. J Antimicrob Chemother. 2015 Nov;70(11):2992-9. doi: 10.1093/jac/dkv220. PubMed PMID: 26221017.

29. Rajabally N, Kullin B, Ebrahim K, et al. A comparison of *Clostridium difficile* diagnostic methods for identification of local strains in a South African centre. J Med Microbiol. 2016 Feb 9;65(4):320-327. doi: 10.1099/jmm.0.000231. PubMed PMID: 26860329.

30. Kullin B, Wojno J, Abratt V, et al. Toxin A-negative toxin B-positive ribotype 017 *Clostridium difficile* is the dominant strain type in patients with diarrhoea attending tuberculosis hospitals in Cape Town, South Africa. Eur J Clin Microbiol Infect Dis. 2017 Jan;36(1):163-175. doi: 10.1007/s10096-016-2790-x. PubMed PMID: 27696234.
